# A Recombinase Aided Amplification Assay for Rapid Detection of the *Klebsiella pneumoniae* Carbapenemase Gene and Its Characteristics in *Klebsiella pneumoniae*

**DOI:** 10.3389/fcimb.2021.746325

**Published:** 2021-09-20

**Authors:** Weiwei Zhang, Yanling Feng, Hanqing Zhao, Chao Yan, Junxia Feng, Lin Gan, Jinghua Cui, Shiyu Liu, Rui Zhang, Shuheng Du, Nannan Li, Wenjian Xu, Juqiang Han, Rongkuan Li, Guanhua Xue, Jing Yuan

**Affiliations:** ^1^The Second Affiliated Hospital of Dalian Medical University, Liaoning, China; ^2^Department of Bacteriology, Capital Institute of Pediatrics, Beijing, China; ^3^Department of Daily Clinic, Seventh Medical Center of People’s Liberation Army (PLA) General Hospital, Beijing, China

**Keywords:** recombinase-aided amplification, rapid detection, *bla*_KPC_, characteristic, *Klebsiella pneumoniae*

## Abstract

*Klebsiella pneumoniae* carbapenemase genes (*bla*_KPC_) play an important role in carbapenem-resistant *Enterobacteriaceae* in China. A rapid detection method for *bla*_KPC_ genes and investigations into the molecular characteristics of *bla*_KPC_ positive *Klebsiella pneumoniae* were necessary. In this study, an easy and rapid recombinase aided amplification assay (RAA) for *bla*_KPC_ was established. This protocol could be completed at 39°C in 15–20 min. The sensitivity of this assay was determined as 48 copies per reaction, and the specificity was 100%. The *bla*_KPC_ RAA method could be used for clinical diagnosis and epidemiological investigation. Among 801 fecal samples from inpatients, 34 *bla*_KPC_ positive isolates were identified from each sample, of which 23 isolates were *K. pneumoniae*. ST11 with *bla*_KPC-2_ was the most prevalent type. All these strains were multidrug resistant and carried various virulence genes. Fecal carriage of *bla*_KPC_ positive carbapenem-resistant *K.pneumoniae* poses significant challenges for public health control.

## Introduction

Carbapenem antibiotics have once been regarded as the last line of defense for the treatment of resistant *Enterobacteriaceae* bacteria. In recent years, with the extensive application of carbapenem antibiotics, carbapenem-resistant *Enterobacteriaceae* (CRE) have shown a steadily increasing trend (Xu et al., 2015). *Klebsiella pneumoniae* carbapenemase (KPC) is one of the significant carbapenemases that belong to class A of the Ambler classification system, and they are capable of hydrolyzing carbapenems and β-lactamase. Rectal colonization or carriage of KPC serves as an important reservoir for dissemination of resistant bacteria (Borer et al., 2012). This could result in both horizontal and vertical spreading between and within species. Therefore, a rapid and simple detection method is integral to the control of KPC dissemination during clinical diagnosis and treatment.

At present, molecular detection methods for *bla*_KPC_ are slow and laborious. Recombinase aided amplification assay (RAA) is a novel isothermal amplification technology that uses specific enzymes and proteins for DNA amplification at 39°C within 15–20 min. In the RAA assay, the three major proteins including single strand DNA binding protein, recombinase UvsX (anneals the primers to the template DNA), and DNA polymerase (for amplification and extension) make the reaction system rapid and specific. Recently, RAA has been successfully used to detect various microbial pathogens (Zhang et al., 2017; Qin et al., 2021), but it has not been used to detect resistance genes.

In this study, we established an RAA detection method to accurately identify the *bla*_KPC_ gene. In addition, as KPC positive species in fecal samples are frequently encountered, approximately 90%–100% in *K. pneumoniae* (*Kpn*) (Liu et al., 2019; Pan et al., 2019; Xu et al., 2020), we further analyzed the characteristics of KPC positive *K. pneumoniae* including multilocus sequence typing (MLST), serotype, mucus phenotype, and virulence genes. Our results provided a rapid clinical screening test to improve the control of *K. pneumoniae*.

## Materials and Methods

### Clinical Samples, Strain Collection, and DNA Extraction

A total of 801 nonduplicate stool specimens for routine analysis were randomly collected from 434 adult inpatients in a general hospital and 367 pediatric inpatients in a pediatrics hospital in Beijing, China from December, 2020 to February, 2021. The two hospitals were both tertiary comprehensive hospitals. Clinical information for these isolates was simultaneously collected.

The collected specimens were processed in two parts. A 200 mg sample was taken from each fecal sample for total DNA extraction, according to the instructions of the QIAamp DNA Mini Kit (Qiagen, Hilden, Germany). The remaining specimens were cultured on MacConkey Agar plates. The isolated strains were verified using a VITEK-2 compact system (bioMerieux, Marcy l’Etoile, France) and 16s-rRNA Sequencing (Sangon Biotech Co., Ltd., Shanghai, China).

### Primers and Probe Design for RAA

**Figure 1:**
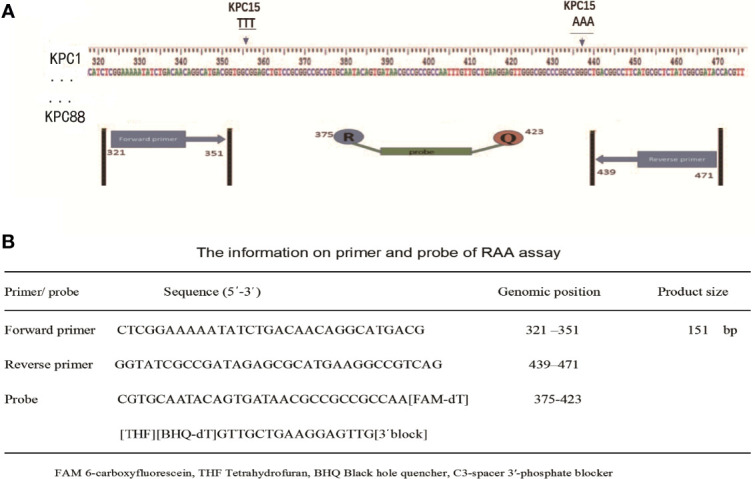
Selection of specific regions and primer positions. **(A)** A total of 87 *bla*_KPC_ genes (*bla*_KPC-1_ to *bla*_KPC-82_ and *bla*_KPC-84_ to *bla*_KPC-88_) were released in the NCBI website, and *bla*_KPC83_ was not released. The primers and probe sets were designed within the highly conserved regions of 87 *bla*_KPC_ genes (320-472 bp region) by avoiding two mutation regions of *bla*_KPC-15_. **(B)** The primer and probe sequences used for RAA assays.

A total of 87 *bla*_KPC_ genes were present in the NCBI website, all these genes were downloaded for the primer design. The conserved regions of 87 *bla*_KPC_ genes were aligned and contrasted using BioEdit Sequence Alignment Editor 7.2.1 software. The primers and probe were manually designed within the highly conserved regions (**Figure 1**), according to the principles of RAA primer and probe design. NCBI Primer-BLAST was used to check and ensure the specificity of the primers and probe. Oligo7 software was used to carefully analyze dimer formation among themselves (self-dimers) and the formation of secondary structures (hairpins). The primers and probe were synthesized by Sangon Biotech Co., Ltd. (Shanghai, China).

### RAA Assay

The full-length *bla*_KPC-2_ gene (*Kpn* ATCC1705) was PCR-amplified and cloned into vector pUC57 (Tiangen Biotech Co., Ltd, Beijing, China). A dilution series with the recombinant plasmids (ranging from 10^7^ to 10^0^ copies/μl) was prepared to evaluate the sensitivity of the RAA assay.

The RAA amplification assays were performed in a 50 μL reaction volume using a commercial RAA kit (Qitian Biological Co., Ltd., Jiangsu, China). The reaction mixture contained 2 μL of extracted DNA template, 25 μL of reaction buffer, 15.7 μL of DNase-free water, 2.1 μL of primer F, 2.1 μL of primer R, 0.6 μL of probe, and 2.5 μL of magnesium acetate. The tubes with the reaction mixture containing the RAA enzyme mix in a lyophilized form, were placed into a B6100 Oscillation mixer (QT-RAA-B6100, Qitian Bio-Tech Co. Ltd., Jiangsu, China) and incubated for 4 min, mixed briefly, centrifuged, and finally transferred to a fluorescence detector (QT-RAA-1620, Qitian Bio-Tech Co. Ltd., Jiangsu, China) to be measured for 10–20 min at 39°C. A positive control (*bla*_KPC_ positive plasmid) and negative control (double-distilled water) were included in each run.

### Analytical Sensitivity, Specificity, and Reproducibility of the RAA Assay

Analytical sensitivity of the RAA assay was carried out using a serial diluted recombinant plasmid ranging from 10^7^ to 10^0^ copies/μL. The specificity assay was evaluated by the testing of *Kpn* ATCC1705 (carrying *bla*_KPC-2_), *Acinetobacter baumannii* (carrying *bla*_KPC-3_), *Pseudomonas aeruginosa* (carrying *bla*_KPC-2_), and another 12 bacterial samples without *bla*_KPC_ genes (PCR verified, data not shown), as follows: *Kpn* ATCC2146 (carrying *bla*_NDM-1_), *K. oxytoca* (carrying *bla*_IMP-4_), *Escherichia coli* (carrying *bla*_OXA-48_), *Shigella sonnei*, *Salmonella enteritidis*, *Proteus mirabilis*, *Yersinia enterocolitica*, *Campylobacter jejuni*, *Citrobacter freundii*, *Burkholderia cepacia*, *Haemophilus influenzae* and *Morganella morganii*. All the above mentioned clinical strains were originally preserved at the Department of Bacteriology of Capital Institute of Pediatrics. In addition, six replicates were performed on six different days to validate the reproducibility of the RAA assay.

### PCR Detection and Sequencing

There was currently no commercial kit for *bla*_KPC_ detection, therefore standard PCR was used for the reference. A 50 μL reaction volume containing the following components was used for all the PCRs: 25 μL of PCR Master Mix reagent (Tiangen Biotech Co., Ltd, Beijing, China), 19 μL of double-distilled water, 2 μL of 10 μM KPC-F primer (5′-ATGTCACTGTATCGCCGTCT-3′) and KPC-R primer (5′-TTACTGCCCGTTGACGC-3′), and 2 μL of DNA template. PCR products were sequenced at Sangon Biotech Co., Ltd. (Shanghai, China) and compared to the NCBI database (http://blast.ncbi.nlm.nih.gov).

### Detection and Evaluation of the RAA Assay With Clinical Samples

All the *bla*_KPC_-positive samples and the randomly selected *bla*_KPC_-negative samples that were detected by standard PCR from 801 samples were used to validate the performance of the established RAA method. The results of the RAA assay were compared with those from a standard PCR assay.

### Molecular Typing Analyses of the KPC Positive *Kpn* Isolates

MLST and KL serotyping were conducted to phylogenically classify the KPC positive *Kpn*. Seven housekeeping genes of *Kpn* were amplified and sequenced based on protocols as described (Diancourt et al., 2005). The *wzi* gene for each strain was amplified with specific primers as reported previously (Brisse et al., 2013). Sequence types (ST) and KL-genotypes were identified using the online database at the Pasteur Institute Multilocus Sequence Typing website for *Kpn* (https://bigsdb.pasteur.fr).

### Antimicrobial Susceptibility Testing of the KPC Positive *Kpn*

Antimicrobial susceptibility testing was performed using the VITEK2 compact system. The minimal inhibitory concentration (MIC) values for co-trimoxazole, aztreonam, ampicillin/sulbactam, piperacillin/tazobactam, cefazolin, cefuroxime, ceftazidime, cefoperazone/sulbactam, cefotaxime, cefepime, gentamicin, amikacin, ciprofloxacin, levofloxacin, tetracycline, imipenem, and meropenem were determined. *Escherichia coli* ATCC25922 and *Pseudomonas aeruginosa* ATCC27853 were used for quality control. The results were recorded, according to the 2020 Clinical Laboratory Standards Institute’s threshold.

### Hypermucoviscosity Phenotype, Growth Curve, and Virulence Genes Assay of the KPC Positive *Kpn*

The string test of strains was performed to determine the hypermucoviscosity phenotype, as previously described (Shon et al., 2013). Growth curve assays for a randomly selected isolate of each type of *Kpn* were conducted to assess the *in vitro* fitness of strains for growth kinetics analysis. Experiments were repeated in triplicate. The growth curve results were calculated by statistical analysis and the average absorbance values were used for drawing. Various virulence genes involved in invasion infection and colonization (*rmpA2*, *rmpA*, *magA*, *fimH*, *wabG*, *uge*, *allS*, *mrkD*, *aerobactin*, *wcaG*, *kfuBC*, *iucA*, *iroN*, *ybtA*, *ureA*, *iucB*, *entB*, and *iroB*) were amplified, which were related to capsular polysaccharide synthesis, fimbriae synthesis, iron acquisition system, urease, and lipopolysaccharide. Carrier rates of virulence factors grouped by the differing MLST and KL-type were documented.

### Statistical Analysis

Statistical analyses of the kappa and P-values of the RAA and standard PCR assays (with sequencing), were calculated with SPSS 21.0 (IBM, Armonk, NY, USA). The trials were performed in triplicate.

## Results

### Primers and Probe Design of the RAA Assay

As shown in **Figure 1A**, the RAA primers and probe for the *bla*_KPC_ gene were manually designed within the conserved regions of the 87 *bla*_KPC_ types, and alignment among the different *bla*_KPC_ types was presented. Sequences for two optimal primers and one probe were listed in **Figure 1B**.

### Analytical Sensitivity and Specificity of the RAA Assay

**Figure 2:**
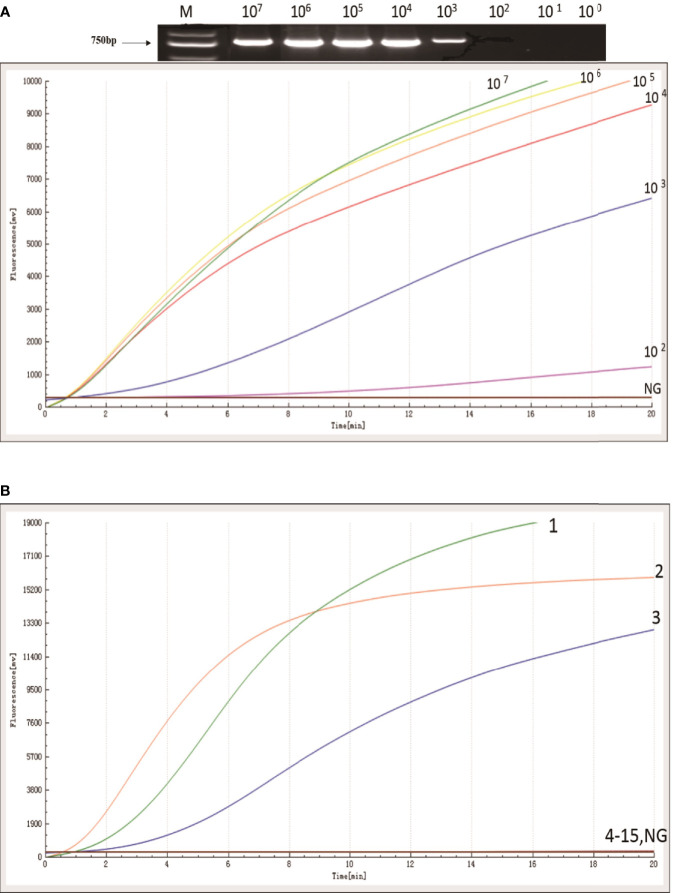
Sensitivity and specificity of RAA assay. **(A)** An increase in the fluorescence signal was observed from 1× 10^7^ to 1 × 10^2^ copies/reaction. **(B)** *Kpn* ATCC1705, *Acinetobacter baumannii*, and *Pseudomonas aeruginosa* produced amplification signals (1, 2, and 3), while the other KPC negative bacterial strains (4-15) and negative control were negative.

The sensitivity of the RAA assay for *bla*_KPC_ was shown in **Figure 2A**. An increase in the fluorescence signal was observed from 10^2^ to 10^7^ copies/uL. The detection limit of the RAA assay was 48 copies per reaction while standard PCR was 480 copies per reaction. RAA is more sensitive when compared with standard PCR.

The specificity of the RAA assay for *bla*_KPC_ was confirmed by testing three *bla*_KPC_-positive samples and another 12 *bla*_KPC_-negative samples. In contrast with the other *bla*_KPC_-negative bacterial samples and the distilled water control (4 to 15, in **Figure 2B**), only *Kpn* ATCC1705, *Acinetobacter baumannii*, and *Pseudomonas aeruginosa* produced amplification signals (1, 2, and 3, in **Figure 2B**); meanwhile, no cross-reactivity was observed for other types of carbapenemase gene. The RAA assay for the detection of *bla*_KPC_ was specific (100%).

### Evaluation of the RAA Assay Using Clinical Samples

Initially, during the study period, 801 stool samples were used for screening *bla*_KPC_ genes by standard PCR, of which 34 (4.2%, 34/801) samples were positive for *bla*_KPC_. Subsequently, all 34 *bla*_KPC_-positive samples and 100 randomly selected *bla*_KPC_-negative samples were used for RAA validation. The results of the RAA assay were in complete agreement with those obtained with standard PCR. No false-positives were found between the RAA assay and the standard PCR, as shown in **Table 1**.

**Table 1:** Detection of *bla*_KPC_ in clinical specimens.

			RAA			Kappa	P-value
			Positive	Negative	Total		
**Standard PCR**	**Adult**	positive	21	0	21	1	<0.001
		negative	0	50	50		
		Total	21	50	71		
	**Children**	positive	13	0	13	1	<0.001
		negative	0	50	50		
		Total	13	50	63		

### Clinical Characteristics of *bla*_KPC_-Positive Strains, MLST, and KL-Serotype

The carbapenem resistant strains were isolated from all the 34 *bla*_KPC_-positive samples that were detected by standard PCR and RAA. Of them, 23 *Kpn* (67.6%, 23/34) were identified, which referred to 2.9% (23/801) in fecal samples. All the *bla*_KPC_-positive *Kpn* strains carried *bla*_KPC-2._ Characteristics, including age, gender, ward distribution, and sequencing results for the 23 cases were shown in **Table 2**. Overall, most of the *bla*_KPC_-positive *Kpn* cases (20/23) came from ICU wards and surgical wards.

**Table 2:** Summary of *bla*_KPC_ positive *Kpn* isolates.

Patients	Isolates Number	Ward	Sex (age: y/mo)*	String test	Molecular typing	Sequencing
**Adults**	Kpn-A1	ICU	F (72 y)	–	ST11-KL25	*bla*_KPC-2_
	Kpn-A2	ICU	M (86 y)	–	ST11-KL25	*bla*_KPC-2_
	Kpn-A3	ICU	M (70 y)	–	ST11-KL10	*bla*_KPC-2_
	Kpn-A4	ICU	M (87 y)	–	ST11-KL10	*bla*_KPC-2_
	Kpn-A5	ICU	M (92 y)	–	ST11-KL10	*bla*_KPC-2_
	Kpn-A6	Respiratory	M (94 y)	+	ST11-KL10	*bla*_KPC-2_
	Kpn-A7	Emergency	M (80 y)	–	ST11-KL10	*bla*_KPC-2_
	Kpn-A8	ICU	M (80 y)	–	ST11-KL10	*bla*_KPC-2_
	Kpn-A9	Gastrointestinal surgery	M (40 y)	–	ST11-KL10	*bla*_KPC-2_
	Kpn-A10	Neurology ICU	F (75 y)	–	ST11-KL10	*bla*_KPC-2_
	Kpn-A11	Acute abdomen surgery	M (32 y)	+	ST11-KL10	*bla*_KPC-2_
	Kpn-A12	ICU	M (78 y)	+	ST11-KL47	*bla*_KPC-2_
	Kpn-A13	ICU	M (80 y)	+	ST147-KL64	*bla*_KPC-2_
	Kpn-A14	Acute abdomen surgery	M (50 y)	–	ST147-KL64	*bla*_KPC-2_
**Children**	Kpn-C1	ICU	M (13 mo)	–	ST11-KL47	*bla*_KPC-2_
	Kpn-C2	Neonatal surgery	M (2 mo)	–	ST11-KL47	*bla*_KPC-2_
	Kpn-C3	Neonatal surgery	F (2 mo)	–	ST11-KL47	*bla*_KPC-2_
	Kpn-C4	Neonatal surgery	M (0.3 mo)	–	ST11-KL47	*bla*_KPC-2_
	Kpn-C5	ICU	M (1 mo)	–	ST11-KL47	*bla*_KPC-2_
	Kpn-C6	ICU	M (0.7 mo)	–	ST11-KL47	*bla*_KPC-2_
	Kpn-C7	ICU	F (4 mo)	–	ST11-KL47	*bla*_KPC-2_
	Kpn-C8	Neurology	M (6 mo)	–	ST11-KL47	*bla*_KPC-2_
	Kpn-C9	ICU	F (5 mo)	–	ST11-KL47	*bla*_KPC-2_

*The age unit for adults is year (y), and the age unit for children is month (mo). ICU, intensive care unit.

MLST analysis revealed that ST11 was the predominant ST (91.3%, 21/23), followed by ST147 (8.7%, 2/23). We further investigated the KL serotypes of these strains: KL47 (43.5%, 10/23), KL10 (15.6%, 9/23), and KL25 (8.7%, 2/23). There were three KL serotypes in ST11; ST11-KL47 were the most common type (10/21), followed by ST11-KL10 (9/21), ST11-KL25 (2/21), and ST147-KL64 (2/21) (**Table 2**).

Interestingly, a male ICU patient (Kpn-A13 in **Table 2**) diagnosed with colon cancer, had a sputum culture result that was carbapenem-resistant *Kpn*, following clinical laboratory tests. We isolated the *bla*_KPC_-positive *Kpn* stain from his stool sample and sputum sample, and they shared the same genotype (ST147-KL64).

### Antimicrobial Susceptibility of *bla*_KPC_-Positive *Kpn* Isolates

Antimicrobial-susceptibility testing revealed that all *bla*_KPC_-positive *Kpn* isolates were resistant to Carbapenem antibiotics (Imipenem and Meropenem, 100%) and Beta-lactam antibiotics (aztreonam, piperacillin/tazobactam, cefazolin, ceftazidime, cefoperazone/sulbactam, cefepime; 100%) (**Supplementary Figure S1**); furthermore, they were also multidrug resistant. However, 95.7% of these isolates were susceptible to Tigecycline (22/23) and 52.2% retained susceptibility to Sulfonamides antibiotics (12/23).

### Hypermucoviscosity Phenotype, Growth Curve, and Virulence Genes of *bla*_KPC_-Positive *Kpn* Isolates

**Figure 3:**
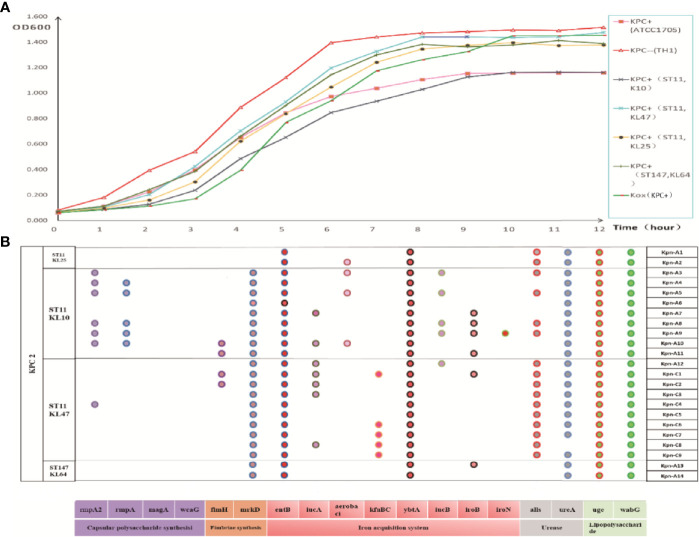
**(A)** Growth curve of each group of strains. The data exhibited are the average absorbance values of three independent experiments. **(B)** Distribution of virulence gene in strains. The presence of virulence genes in a specific genome is indicated by different colored circles. Virulence genes and their roles are shown at the bottom.

The string test showed that four strains were hypermucoviscous (**Table 2**), which belonged to ST11(75%) and ST147 (25%), respectively. The *bla*_KPC_-positive strains were in a logarithmic growth phase for 1–8 hours, reached a maximum at ~8 hours, and then maintained a relatively stable state (**Figure 3A**). There was no statistically significant difference in growth of *bla*_KPC_-positive and *bla*_KPC_-negative strains (P > 0.05).

As shown in **Figure 3B**, *bla*_KPC_-positive *Kpn* strains contained multiple virulence genes including *entB* (100%, iron acquisition system), *uge* (100%, lipopolysaccharide), *ybtA* (100%, iron acquisition system), *wabG* (100%, lipopolysaccharide), *mrkD* (91%, fimbriac synthesis), *alls* (70%, urease), and *ureA* (96%, urease). ST11-KL10 strains harbored almost all the virulence genes. ST11 strains (48%, 183/378) carried more virulence genes than ST147 strains (36%, 13/36).

## Discussion

The *bla*_KPC_ gene is a carbapenem resistance gene that is located in mobile transposon Tn*4401*, which facilitates its transfer between plasmids, species, patients, and different countries (Munoz and Quinn, 2009). The KPC epidemic is problematic for global health. Therefore, identifying and screening *bla*_KPC_ genes in clinical settings would be extremely helpful.

In the present study, we established an RAA assay for the detection of the *bla*_KPC_ gene. The primers and a probe were designed in the conserved region of all 87 existing *bla*_KPC_ genes, thus allowing the amplification of all these genes. This assay, therefore, had good specificity. The sensitivity of the *bla*_KPC_ RAA assay was superior to standard PCR and reached the same level as other nucleic acid detections for *bla*_KPC_, such as real-time PCR (5–40 copies/reaction) (Chen et al., 2011; Subirats et al., 2017) and loop-mediated isothermal amplification assay (LAMP) (40-10^5^CFU/mL) (Nakano et al., 2015; Srisrattakarn et al., 2017). The agreement between the RAA and standard PCR assays was 100%, which suggested that the RAA assay was of sufficient quality to be used for clinical *bla*_KPC_ screening.

RAA assay was superior to LAMP or real-time PCR assays, in terms of the simplicity, rapidity and cost (Shen et al, 2019). Real-time PCR requires varying cycling temperatures under rigorous conditions, while RAA assay only need a constant 39°C simple device. In addition to unsophisticated laboratory setting, RAA assay could be completed in 15-20 min, while real-time PCR requires 2-3 hours, which represents a significant reduction of the turnaround time. Real-time PCR is difficult to perform in laboratories with limited funds, poor equipment, and a shortage of technicians. As regards the cost of assay, each test for LAMP assay costs 5-6 $. the RAA cost for one sample is about half of that by LAMP. The high cost of LAMP assays restricts its use in clinical settings.

In the present study, the most common *bla*_KPC_ genotype (*bla*_KPC-2_) and the fecal carriage rate were consistent with the other studies (Zhao et al., 2014; Liu et al., 2019). Also, we found *bla*_KPC_ positive patients mainly in intensive care units (ICU) and surgical wards, where patients received more invasive treatment and numerous antibiotics (including carbapenem), which may lead to the spreading of *bla*_KPC_. Some reports showed that carbapenem treatments led to the emergence of carbapenem-resistant *K. pneumoniae* (CR-*Kpn*) in patients’ gastrointestinal tracts, which subsequently caused infections in other body sites (Zhang et al., 2018). Fecal colonization with CRE is a risk factor for bacterial translocation resulting in subsequent endogenous infections, which can also be seen from our research. One male patient hospitalized for colon cancer, gradually developed pneumonia. The same *bla*_KPC_-positive *Kpn* strains (ST147-KL64) were detected from his stool and sputum samples. This implied that CR-*Kpn* can colonize the gastrointestinal tract and cause disease under special conditions. Long-time asymptomatic carriage of CR-*Kpn* in the intestines of adults and children have recently attracted more attention (Lübbert et al., 2014; Ghaith et al., 2019). In this study, most strains carrying *bla*_KPC_ were *Kpn* (67.6%). This was consistent with other studies (Liu et al., 2019). It is therefore very important for us to study the molecular characteristics of these strains.

In the present study, ST11 with *bla*_KPC2_ was the most prevalent type in *bla*_KPC_ positive *Kpn* strains, which was consistent with the other studies (Liu et al., 2019; Pan et al., 2019; Chen et al., 2020). To further investigate the detailed difference in ST11, serotypes were tested and three KL types (KL25, KL47, KL10) were found. Type KL-47 has been reported in other studies in China (Zheng et al., 2020). In addition to ST11, we also isolated two ST147 with KL64 type *Kpn* strains. This was consistent with another Italian study where they found ST147 with KL64 *bla*_KPC_ positive *Kpn* from rectal swab specimens (Arena et al., 2020). Diverse KL types of *Kpn* strains determine different degrees of detection by the innate immune system involving immune clearance (Doorduijn et al., 2016).

Results of the present study illustrated that only 17.4% (4/23) of strains were hypermucoviscous, which is consistent with other studies (10.9%, 6/55) (Zhang et al., 2020). In the current study, these *bla*_KPC_-positive *Kpn* contained multiple virulence genes associated with synthesis or regulation related factors for infection, including capsular polysaccharide synthesis, fimbriae synthesis, iron acquisition system, urease, and lipopolysaccharide. These strains will definitely have an impact and be problematic in the control and prevention of mutual transmissions.

In this study, all *bla*_KPC_-positive *Kpn* isolates were resistant to carbapenems and beta-lactam antibiotics, which revealed that *bla*_KPC_ genotype matched the phenotype. However, most of them were susceptible to tigecycline (95.7%), and some of them retained susceptibility to sulfonamides (52.2%). The CR-*Kpn* infection therapeutic options are limited to tigecycline, colistin, polymyxin B, etc. Effective surveillance and strict infection control strategies should be implemented to prevent the dissemination of CR-*Kpn*.

There were some limitations in this study, like the small number of cases and the short duration for a retrospective study.

## Conclusion

In conclusion, the RAA assay established in this study had a high specificity and sensitivity and provides a simple and reliable method for *bla*_KPC_ detection, which is suitable for application in primary laboratories or clinical practice. In *bla*_KPC_-positive *Kpn* strains from hospitalized patients’ stool samples, ST11 carrying *bla*_KPC-2_ was the most prevalent type. These strains were multidrug resistant and contained various virulence genes. Effective surveillance and control measures are urgently required to control the transmission of *bla*_KPC_-positive strains.

## Data Availability Statement

The original contributions presented in the study are included in the article/**Supplementary Material**. Further inquiries can be directed to the corresponding authors.

## Ethics Statement

Written informed consent was obtained from the individual(s), and minor(s)’ legal guardian/next of kin, for the publication of any potentially identifiable images or data included in this article.

## Author Contributions

JY, GX, and RL designed the study. WZ, YF, HZ, CY, JF, LG, and JC performed the experiments. RZ, SL, SD, NL, WX, and JH analyzed the results. WZ and GX wrote the article. JY, GX, and RL revised it. All authors contributed to the article and approved the submitted version.

## Funding

This work was financially supported by Public service development and reform pilot project of Beijing Medical Research Institute (BMR2019-11), and Research Foundation of Capital Institute of Pediatrics (GZ-2021-06 and CXYJ-2021-04). National Natural Science Foundation for Key Programs of China Grants (82130065), National Natural Science Foundation of China (31370093 82002191 and 32170201) and FENG foundation (FFBR 202103) to JY

## Conflict of Interest

The authors declare that the research was conducted in the absence of any commercial or financial relationships that could be construed as a potential conflict of interest.

## Publisher’s Note

All claims expressed in this article are solely those of the authors and do not necessarily represent those of their affiliated organizations, or those of the publisher, the editors and the reviewers. Any product that may be evaluated in this article, or claim that may be made by its manufacturer, is not guaranteed or endorsed by the publisher.
