# Supplementary figures and images for: A Recombinase Aided Amplification Assay for Rapid Detection of the Klebsiella pneumoniae Carbapenemase Gene and Its Characteristics in Klebsiella pneumoniae
Source: Front Cell Infect Microbiol. 2021 Sep 20;11:746325. doi: 10.3389/fcimb.2021.746325 (PMC8488121; doi:10.3389/fcimb.2021.746325)

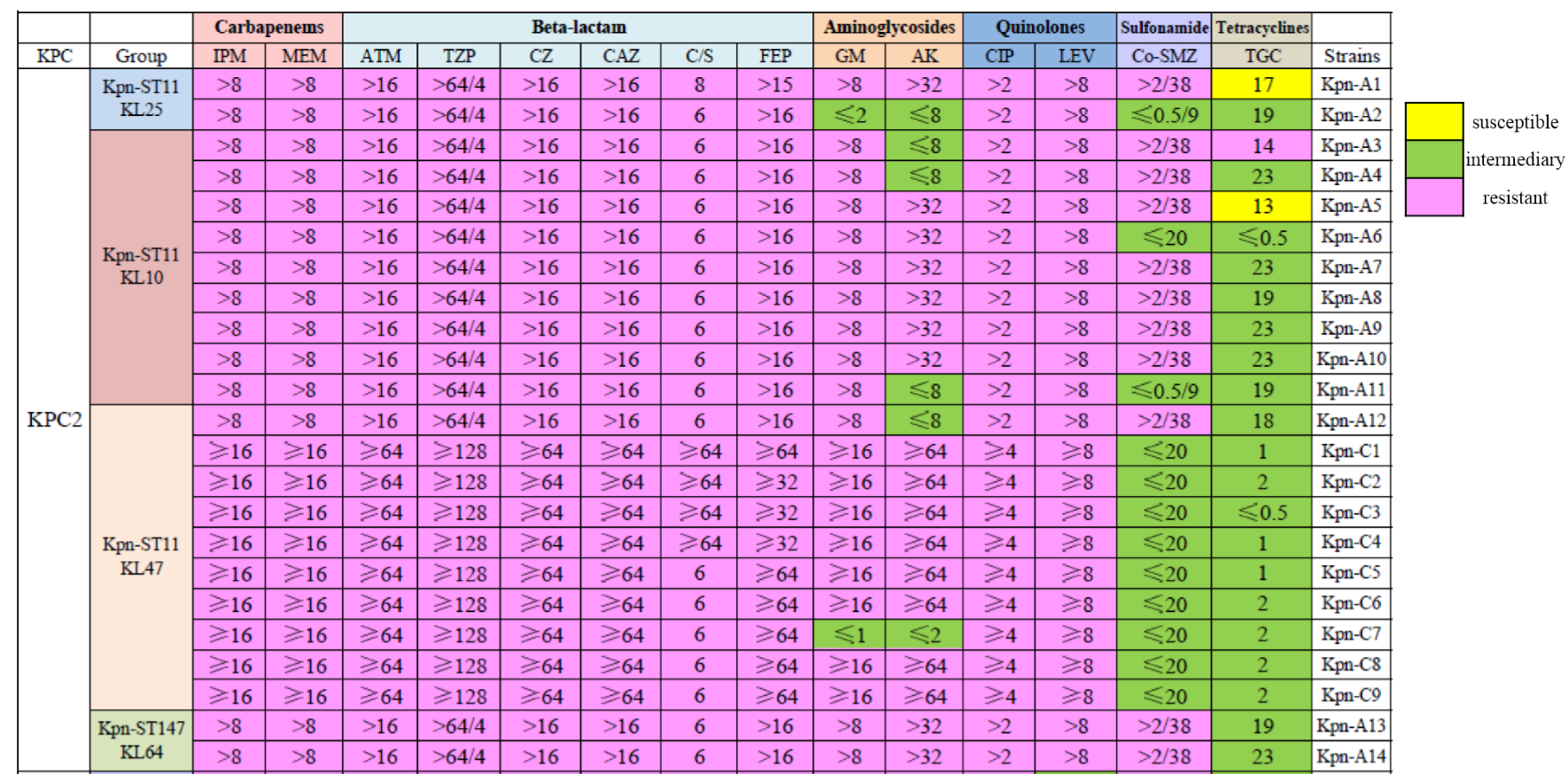

Supplement: Supplementary file 1 [file Image_1.tif]
